# Bis[1,2-bis­(4-fluoro­phen­yl)ethyl­ene-1,2-di­thiol­ato(1−)]nickel(II)

**DOI:** 10.1107/S2056989025007303

**Published:** 2025-08-27

**Authors:** Joseph B. Donahue, Titir Das Gupta, Laura Fiabane, Xiaodong Zhang, James P. Donahue

**Affiliations:** a Saint Paul’s Catholic School, 917 South Jahncke Avenue, Covington, LA 70433, USA; b Department of Chemistry, Tulane University, 6400 Freret Street, New Orleans, Louisiana 70118-5698, USA; Texas A & M University, USA

**Keywords:** crystal structure, di­thiol­ene, radical monoanion, nickel, electron-withdrawing, C—H→F hydrogen bonds

## Abstract

The crystal structure of the title compound, [Ni(C_14_H_8_F_2_S_2_)_2_] (**I**), reveals averaged S—C [1.708 (2) Å] and C—C_chelate_ [1.395 (4) Å] bond lengths that are consistent with radical monoanionic ligands paired with a divalent Ni^2+^ ion. Mol­ecules of **I** associate as dyads *via* inter­molecular Ni⋯S close contacts of 3.396 (2) Å. This close association is enabled by a bending of both di­thiol­ene ligands to the same side and away from the NiS_4_ planar inter­ior such that the angle between the seven atom mean planes defined by each NiS_2_C_2_ ring and the first C atom of each aryl substituent is 22.91 (8)°. These dyads form sheets in the *bc* plane that are held together in part by inter­molecular C—H⋯F hydrogen bonds of 2.47 (4) Å.

## Abstract 2

The title compound crystallizes as closely associated pairs across an inversion center, with near approach enabled by Ni⋯S inter­molecular contacts of 3.396 (2) Å and bending of the di­thiol­ene ligands away from one another.

## 1. Chemical context

Since the mid 1960s, when transition-metal di­thiol­ene complexes first elicited inter­est because their electronic structure descriptions were at variance with classical formalisms (Eisenberg & Gray, 2011 ▸), applications arising from their optical, electrochemical, conducting and magnetic properties have continued to drive fundamental studies. Homoleptic nickel bis­(di­thiol­ene) complexes serve as reversibly bleachable dyes in laser Q-switching systems (Mueller-Westerhoff *et al.*, 1991 ▸) and as optical limiting absorbers (Tan *et al.*, 2000 ▸). Asymmetric Group 10 complexes with an ene-1,2-di­thiol­ate donor and an α-di­thione acceptor function as nonlinear optical materials with potential applications in optical switching devices, signal processing, *etc.* (Deplano *et al.*, 2010 ▸; Artizzu *et al.*, 2022 ▸). Partially oxidized Group 10 complexes with dmit [dmit = 2-thioxo-1,3-di­thiole-4,5-di­thiol­ate(2–)] support superconductivity in the crystalline state, a behavior that is rare for discrete coordination compounds (Cassoux, 1999 ▸; Faulmann & Cassoux, 2004 ▸; Kato, 2004 ▸). Di­thiol­ene complexes sustain a variety of magnetic behaviors in the solid state (Robertson & Cronin, 2002 ▸; Faulmann & Cassoux, 2004 ▸), and they have more recently been investigated as a platform for mol­ecule-based qubits (McGuire *et al.*, 2018 ▸, 2019 ▸). Di­thiol­ene complexes of both nickel (Zarkadoulas *et al.*, 2016 ▸) and cobalt (McNamara *et al.*, 2012 ▸; Letko *et al.*, 2014 ▸) have been reported as highly active electrocatalysts for H_2_-evolution. In this context, the structure of K_2_[Co(S_2_C_2_(C_6_H_4_-4-F)_2_)_2_] has been reported in 2014 (Letko *et al.*, 2014 ▸) and remains the only structurally authenticated coordination compound with this ligand variant. The corresponding charge-neutral nickel compound, although used earlier for the preparation of [((F-4-C_6_H_4_)_2_C_2_S_2_)_2_W(CO)_2_] (Sung & Holm, 2002 ▸) and used in a study of its formation of an adduct with quadricyclane (Kajitani *et al.*, 1989 ▸), has not been characterized structurally. As part of an effort to fully map the range of reduction potentials observed for [Ni(S_2_C_2_Ar_2_)_2_] (Ar = aryl substituent) compounds, we have obtained a crystalline sample of [Ni(S_2_C_2_(C_6_H_4_-4-F)_2_)_2_] and subjected it to an X-ray diffraction study. We detail its structure herein, particularly in contrast to that of [Ni(S_2_C_2_(C_6_H_4_-4-Cl)_2_)_2_].
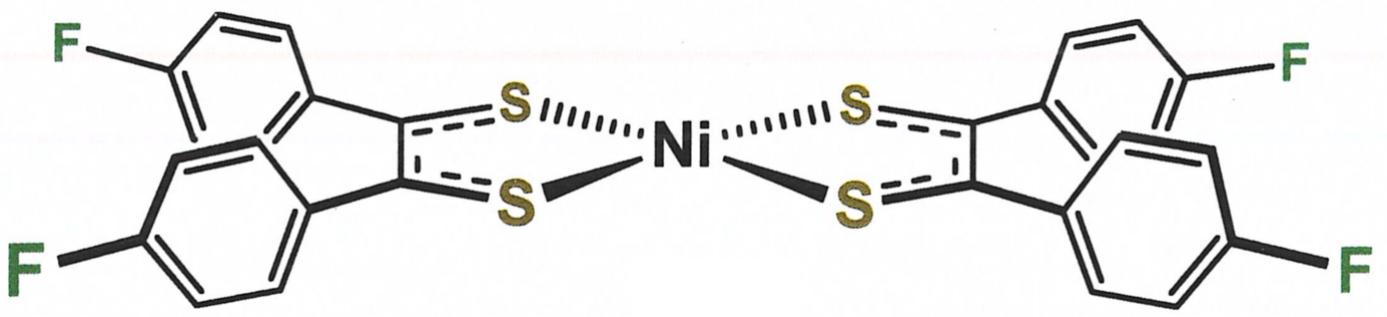


## 2. Structural commentary

**Figure 1:**
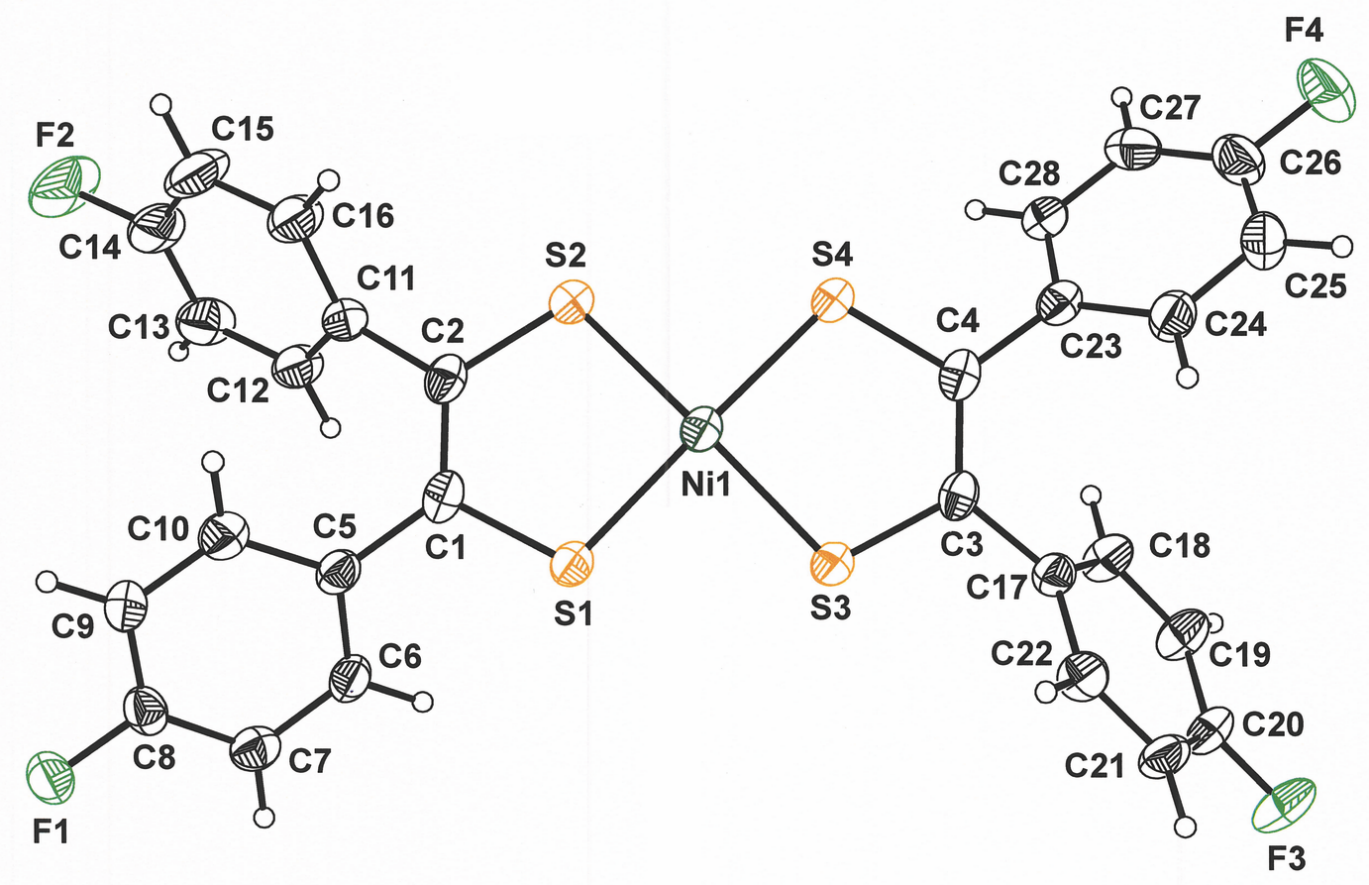
Displacement ellipsoid plot (50% probability) of [Ni(S_2_C_2_(C_6_H_4_-4-F)_2_)_2_] with complete atom labeling.

**Figure 2:**
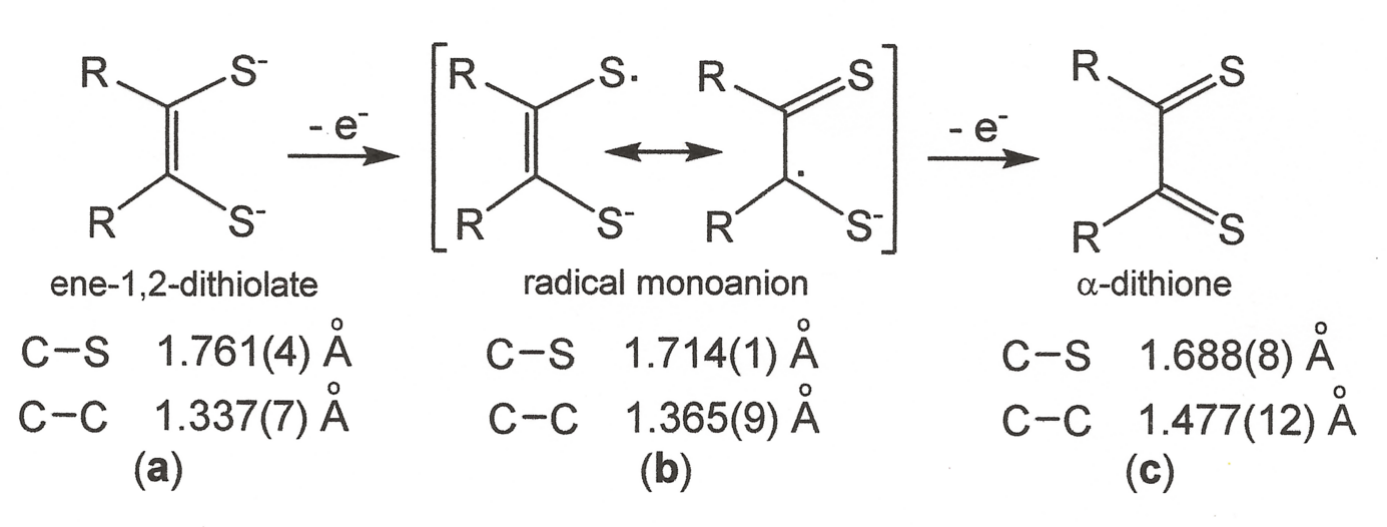
Redox levels of the di­thiol­ene ligand with experimentally determined intra­ligand S—C and C—C bond lengths that are diagnostic of each redox state.

An image of [Ni(S_2_C_2_(C_6_H_4_-4-F)_2_)_2_], **I**, complete with atom labeling and 50% displacement ellipsoids, is presented in Fig. 1 ▸. The averaged S—C and C—C_chelate_ bond lengths are 1.708 (2) and 1.395 (4) Å, respectively, values that are midway between the corresponding inter­atomic distances that have been experimentally established for the fully reduced ene-1,2-di­thiol­ate form (Lim *et al.*, 2001 ▸) and the fully oxidized α-di­thione redox state of the di­thiol­ene ligand (Bigoli *et al.*, 2001 ▸). The di­thiol­ene ligands in **I** are therefore in the half-reduced mono-anionic redox level (Lim *et al.*, 2001 ▸) that provides for charge neutrality when paired with a Ni^2+^*d*^8^ ion (Fig. 2 ▸).

The local geometry around Ni1 is square planar, but a moderate distortion is occasioned by a bending of the two di­thiol­ene ligands to the same side of the central NiS_4_ plane. The angle between the S1–S2–Ni1 and S3–S4–Ni1 planes is 11.62 (7)°, while the angle between the mean planes defined by each NiS_2_C_2_ chelate ring and the first carbon atom of each appended arene ring is double this magnitude at 22.92 (8)°. These differing values and a relatively modest 0.130 Å displacement of Ni1 from the S1–S2–S3–S4 mean plane emphasize that, while the mol­ecule as a whole is bowl-shaped, its bottom is shallow, and the bent character is evident largely because of the peripheral organic groups. The angles formed between the pendant arene rings and the C_2_S_2_ fragment to which they are attached range from 42.7 (1) to 54.1 (1)° and average 47.63 (6)°.

**Figure 3:**
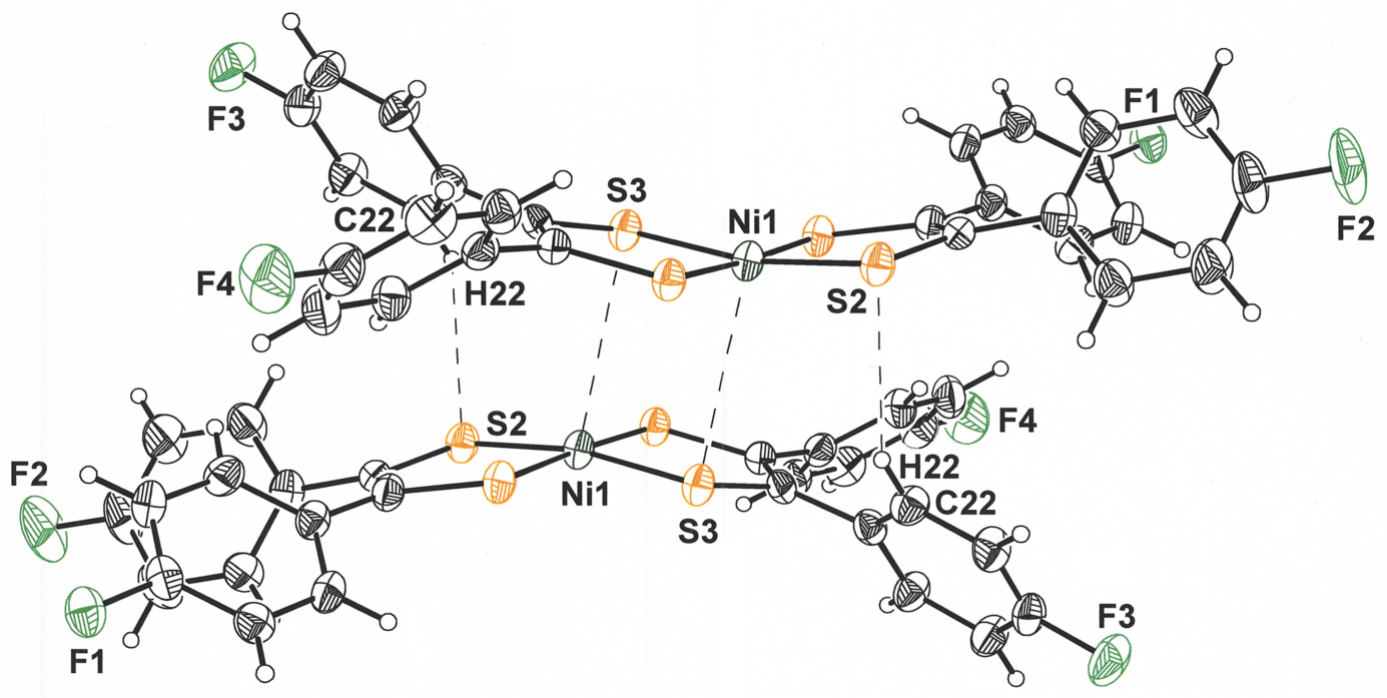
Displacement ellipsoid plot (50% probability) of [Ni(S_2_C_2_(C_6_H_4_-4-F)_2_)_2_] showing its close inter­action with a neighboring mol­ecule across an inversion center.

The bent conformation displayed by **I** is a consequence of close inter­molecular Ni⋯S contacts that place mol­ecules into pairs with a face-to-face, but slightly offset, disposition on either side of an inversion center (Fig. 3 ▸). A rhomboidal shape is defined by this central Ni_2_S_2_ core. The inter­molecular Ni1⋯S3 distance is 3.396 (2) Å, while the Ni1⋯Ni1 distance is 4.106 (1) Å. The former value is substanti­ally less than the 3.8 Å sum of crystallographic radii for Ni (2.0 Å) and S (1.8 Å) (Batsanov, 2001 ▸), therefore implicating it as a decisive inter­action in governing the crystalline packing arrangement. This inter­action is reinforced by a 2.86 (5) Å close contact between S2 of one mol­ecule and H22 of its centrosymmetric counterpart (Fig. 3 ▸). A mononuclear species is pertinent to the solution phase, however, as the ^19^F, ^13^C, and ^1^H NMR spectra show the simpler sets of signals anti­cipated for a *D*_2*h*_-symmetric structure *vs* one with only *C_i_* symmetry.

## 3. Supra­molecular features

**Figure 4:**
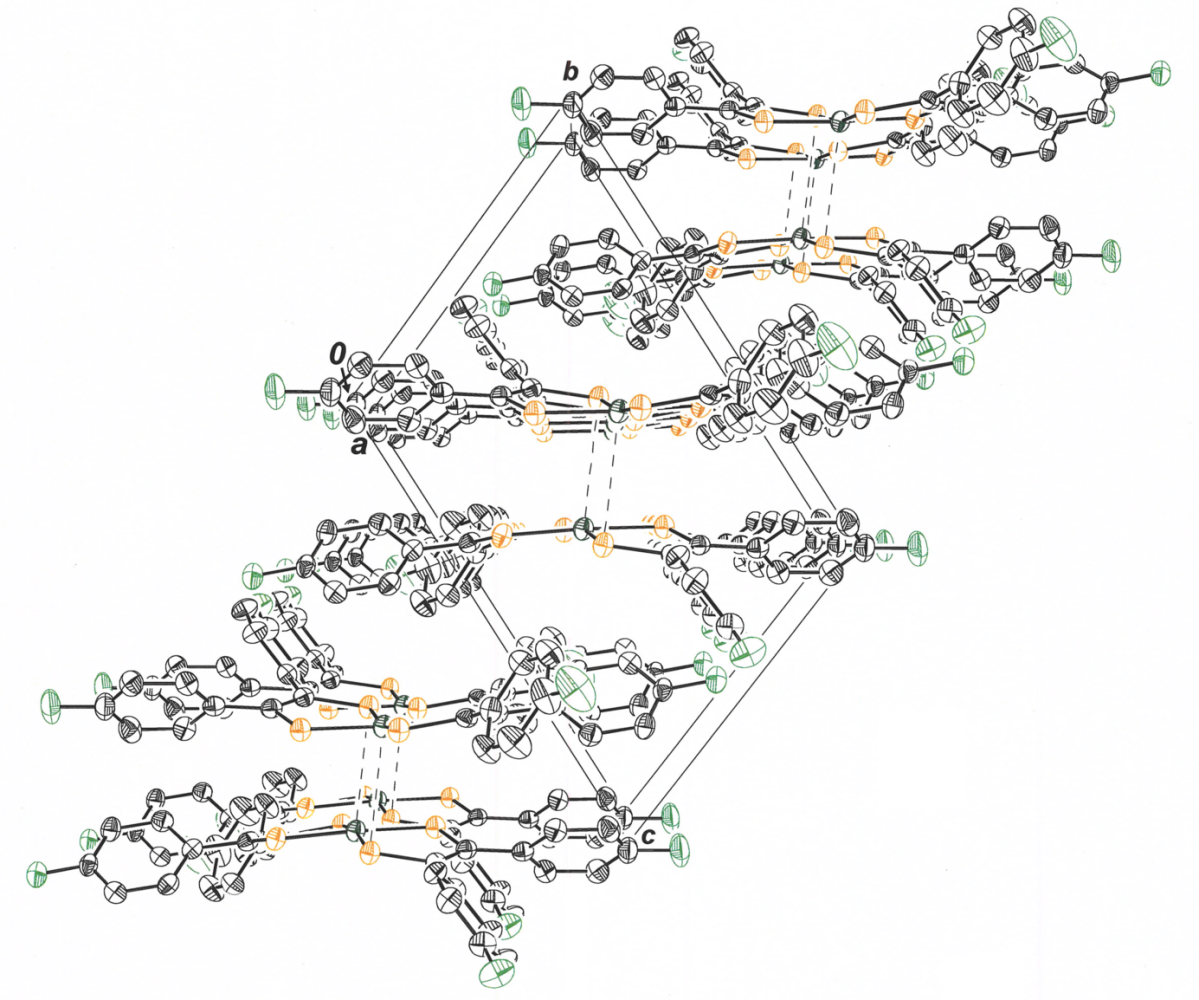
View down the *a* axis of the cell illustrating how the dyads shown in Fig. 2 ▸ are related by translation along the *b* axis. Displacement ellipsoids are shown at 50% probability, and all H atoms are omitted for clarity.

**Figure 5:**
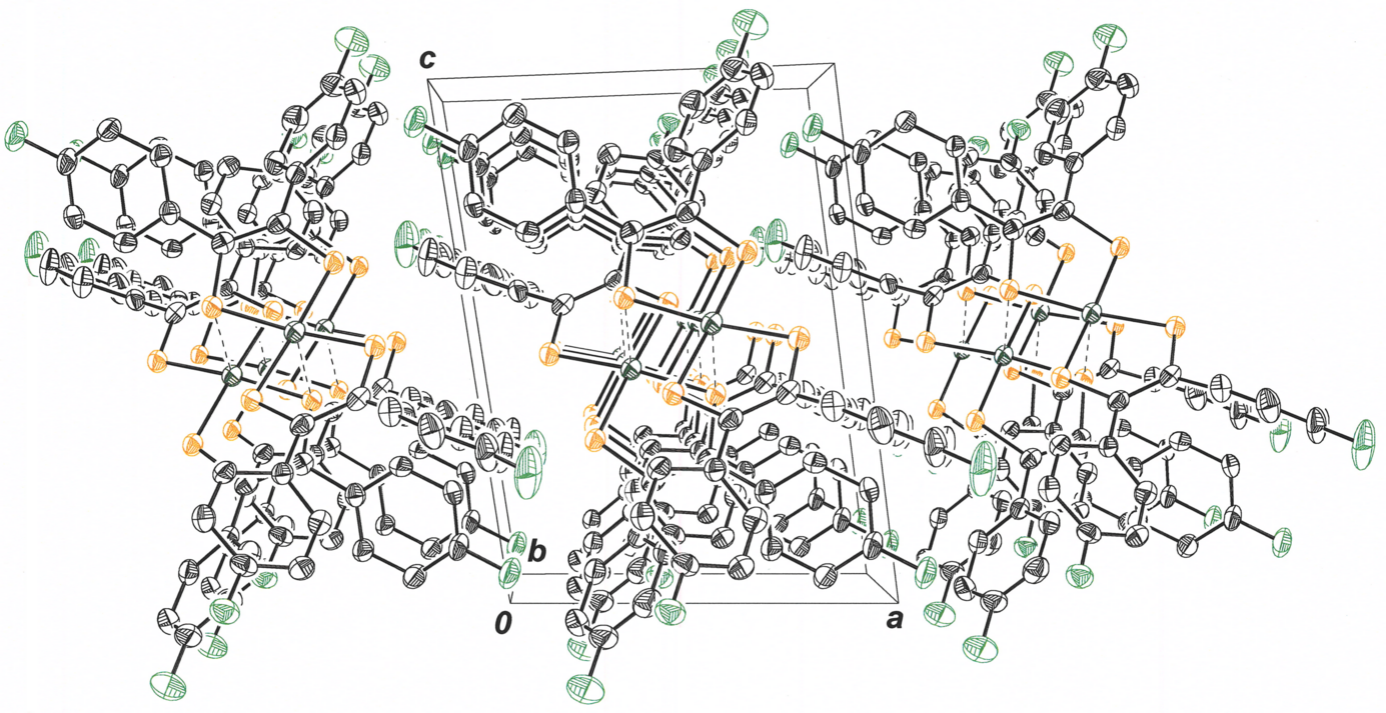
View down the *b* axis of the cell illustrating how the dyads shown in Fig. 2 ▸ form stacks in this axis direction. Displacement ellipsoids are shown at 50% probability, and all H atoms are omitted for clarity.

**Figure 6:**
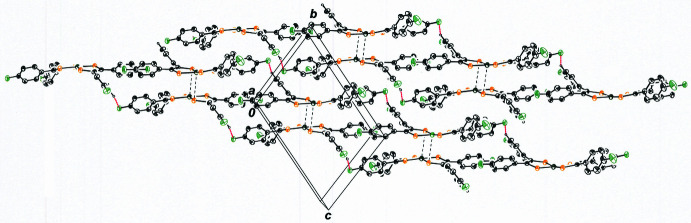
View down the *a* axis of the cell illustrating how the dyads of **I** inter­act in the *bc* plane *via* F⋯C—H hydrogen bonds. All H atoms are omitted except those involved in the F⋯C—H hydrogen bonding. The symmetry operation relating mol­ecules that are participants in a F⋯C—H hydrogen bond is *x*, *y* + 1, *z* + 1. Displacement ellipsoids are presented at the 50% probability level.

**Figure 7:**
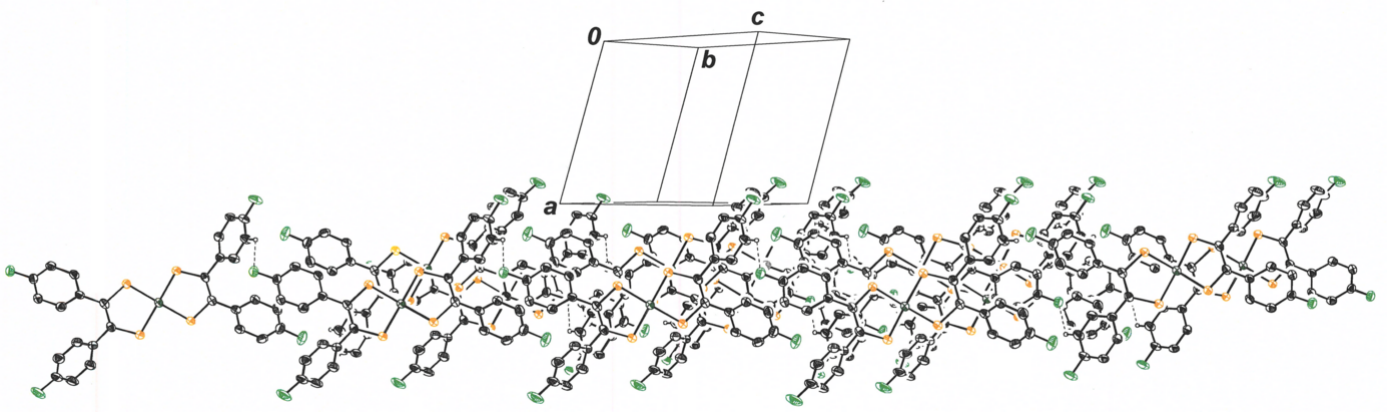
View along the *bc* plane of the packing for **I**, emphasizing the sheet-like arrangement of mol­ecules in this direction. Displacement ellipsoids are shown at the 50% level, and all H atoms are omitted for clarity.

The outward bowing of the di­thiol­ene ligands that enables close approach of the NiS_4_ inter­ior of two mol­ecules provides a concave appearance to the dyadic assembly. These dyads are related by simple translation along the *b* axis of the unit cell (Fig. 4 ▸) such that they eclipse one another in stacks when viewed down the *b* axis (Fig. 5 ▸). Within the *bc* plane, each dyad is held in place by an array of four C—H⋯F hydrogen bonds (Table 1 ▸), with F1 from each mol­ecule in the pair acting as acceptor and C19—H19 from the other ligand of each mol­ecule serving as donor (Fig. 6 ▸). The H19⋯F1 and C19⋯F1 inter­atomic distances are 2.47 (4) Å and 3.136 (5) Å, respectively. The perspective in Fig. 7 ▸ is approximately orthogonal to that in Fig. 6 ▸ and emphasizes the sheet-like arrangement of mol­ecules within the *bc* plane.

## 4. Database survey

**Figure 8:**
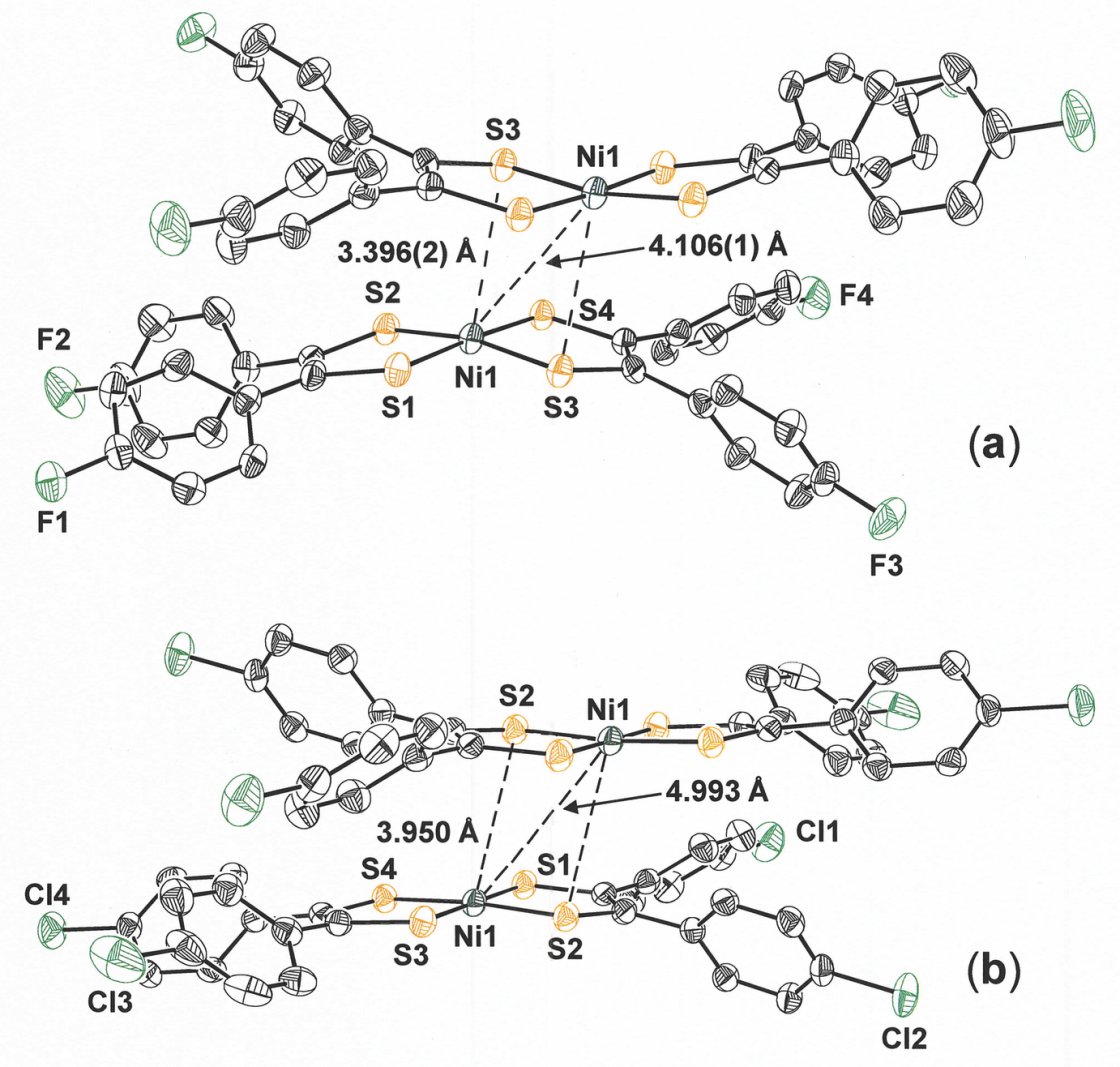
Contrast between the dyadic pairs of [Ni(S_2_C_2_(C_6_H_4_-4-F)_2_)_2_] (**a**) *vs*. [Ni(S_2_C_2_(C_6_H_4_-4-Cl)_2_)_2_] (**b**). Closer association of mol­ecules in (**a**) than (**b**) is enabled by greater bending of the di­thiol­ene ligands away from one another. Displacement ellipsoids are shown at 50% probability, and all H atoms are omitted for clarity.

The arrangement for **I** has qualitative similarity to the fashion in which mol­ecules of [Ni(S_2_C_2_(C_6_H_4_-4-Cl)_2_)_2_] (**II**) are juxtaposed in the crystalline state [Fig. 8 ▸(*b*)] (Koehne *et al.*, 2022 ▸). Pairs of **II** are also disposed around an inversion center in *P*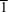, but the degree of bending of the aryl substituents away from one another is somewhat less than in **I**. The angle between the seven atom mean planes defined by the NiS_2_C_2_ chelate rings and the first carbon atom of the arene rings is 11.87 (5)°, approximately half the magnitude of the same distortion in **I**. Because the steric crowding between its Cl-4-C_6_H_4_ substituents is less alleviated by bending away from one another, mol­ecules of **II** associate less closely, with a Ni⋯Ni distance of 4.933 Å and an inter­molecular Ni⋯S distance of 3.950 Å (Fig. 8 ▸). This contrast between **I** and **II** may reflect an attenuated basicity to the di­thiol­ene sulfur atoms in **I**, owing to the greater electron-withdrawing power of F over Cl, such that the Lewis acid character of its Ni^2+^ ion is only fully alleviated by the additional inter­action with a sulfur lone pair from a neighboring mol­ecule.

Other crystallographically characterized nickel bis­(di­thiol­ene) complexes that are symmetrically substituted with aryl groups include [Ni(S_2_C_2_Ph_2_)_2_] (Megnamisi-Belombe & Nuber, 1989 ▸; Kuramoto & Asao, 1990 ▸), [Ni(S_2_C_2_(C_6_H_4_-4-CH_3_)_2_)_2_] (Miao *et al.*, 2011 ▸), [Ni(S_2_C_2_(C_6_H_4_-4-OCH_3_)_2_)_2_] (Arumugam *et al.*, 2007 ▸), [Ni(S_2_C_2_(C_6_H_4_-4-^*t*^Bu)_2_)_2_] (Das Gupta *et al.*, 2023 ▸), and [Ni(S_2_C_2_(C_6_H_3_-3,5-(CH_3_)_2_)_2_] (Das Gupta *et al.*, 2025 ▸). In these cases, such other inter­molecular inter­actions as aryl C—H⋯π_arene_, CH_3_⋯π_arene_, or aryl C—H⋯π NiS_2_C_2_ hydrogen bonds form the basis for packing in the crystalline state rather than Ni⋯S close contacts as in **I**.

**Figure 9:**
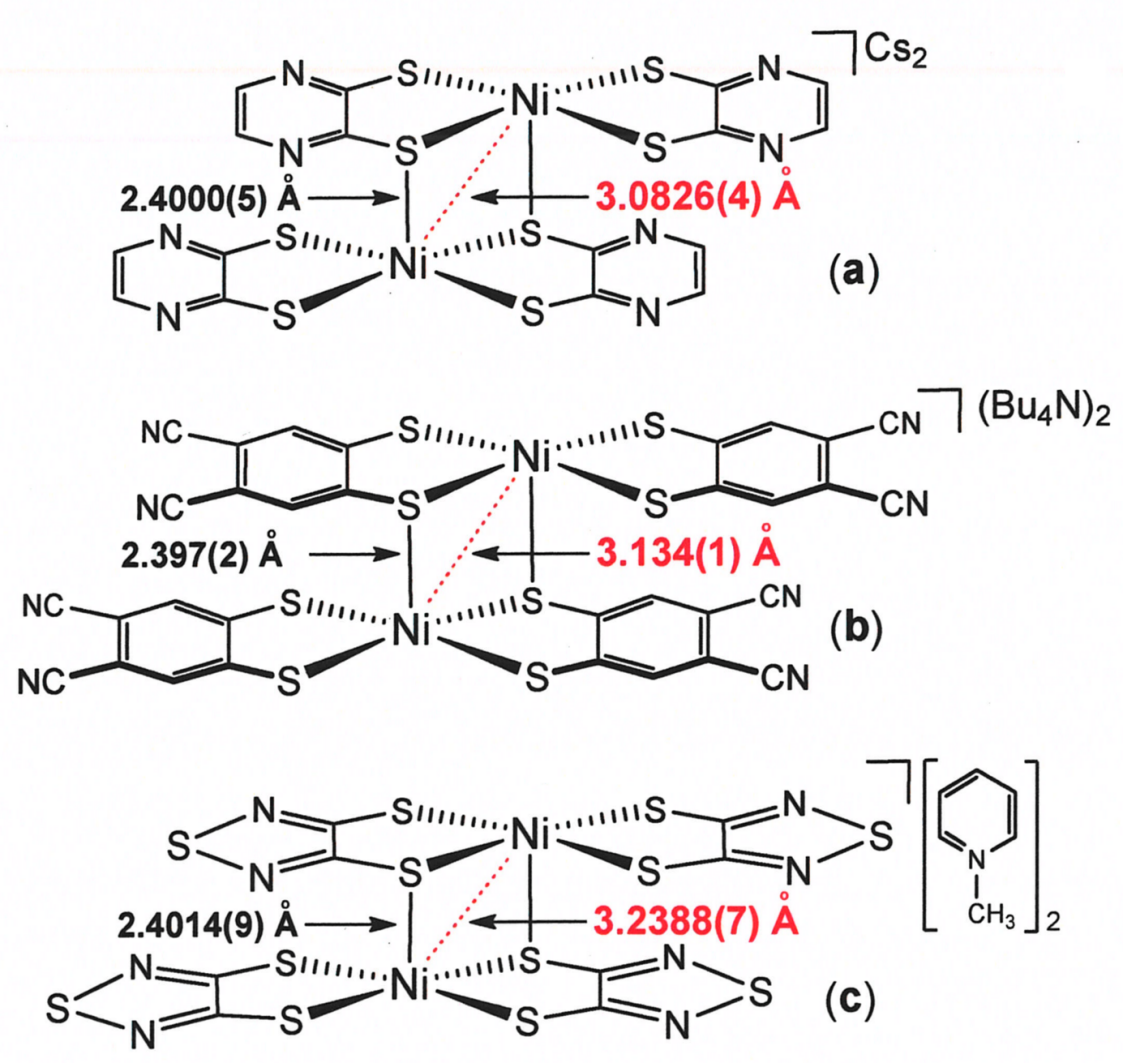
Known dimeric Ni bis­(di­thiol­ene) complexes shown with inter­molecular Ni⋯S and Ni⋯Ni distances.

Although charge neutral diaryl-substituted nickel bis­(di­thiol­ene) complexes other than **I** and **II** do not appear to form paired inter­actions in the crystalline state, anionic nickel complexes with the related pyrazine-2,3-di­thiol­ate (pyzdt) form either stacked monomers or dimers, depending upon the particular identity of the counter-cation (Takaishi *et al.*, 2013 ▸). With Cs^+^, dimeric [[Ni(pyzdt)_2_]_2_]^2−^ prevails with an Ni⋯Ni separation of 3.0826 (4) Å and an inter­molecular Ni⋯S distance of 2.4000 (5) Å (Fig. 9 ▸). Similarly, nickel complexes with 4,5-di­cyano­benzene-1,2-di­thiol­ate (dcbdt) (Simão *et al.*, 2001 ▸) and 1,2,5-thia­diazole-3,4-di­thiol­ate (tdas) (Chen *et al.*, 2016 ▸) form dianionic dimers with bridging Ni⋯S and inter­atomic distances that are the same as in [[Ni(pyzdt)_2_]_2_]^2−^ within experimental error {Ni⋯S: 2.397 (2) Å in [[Ni(dcbdt)_2_]_2_]^2−^, 2.4014 (9) Å in [[Ni(tdas)_2_]_2_]^2−^}. However, the Ni⋯Ni separations vary substanti­ally from that in [[Ni(pyzdt)_2_]_2_]^2−^ {3.134 (1) Å in [[Ni(dcbdt)_2_]_2_]^2−^ and 3.2388 (7) Å in [[Ni(tdas)_2_]_2_]^2−^} because the Ni⋯S distances within the mononuclear fragments of these several complexes differ somewhat. In all these instances, the strong dimeric inter­action is driven by anti­ferromagnetic coupling of the radical monoanionic fragments (Fig. 2 ▸) rather than by presumed Lewis acid–base pairing as in **I**.

## 5. Synthesis and crystallization

The procedure followed was a modification of that described by Mayweg & Schrauzer (1965 ▸). An oven-dried 100 mL Schlenk flask was charged with P_4_S_10_ (3.502 g, 7.88 mmol), 1,2-bis­(4-fluoro­phen­yl)ethane-1,2-dione (2.010 g, 8.16 mmol), and 20 mL of dry dioxane. This mixture was placed under an N_2_ atmosphere with a series of rapid evacuations and backfills and then was vigorously refluxed for 3 h. After cooling to ambient temperature, the heterogeneous mixture was filtered under N_2_*via* filter cannula to afford an amber-colored filtrate. A solution of [Ni(OH_2_)_6_]Cl_2_ (1.001 g, 4.21 mmol) in degassed, deionized H_2_O (20 mL) was transferred to this filtrate, and the mixture was again refluxed with stirring for 3 h. The dark reaction mixture was slowly cooled to ambient temperature overnight. The dark precipitate that formed was collected by vacuum filtration on a Hirsch funnel and washed with portions of H_2_O (2 × 10 mL), MeOH (2 × 10 mL), and Et_2_O (2 × 10 mL). After drying overnight, **I** was obtained in the form of a dark powder. Yield: 0.684 g, 1.11 mmol, 27.2%. *R*_*f*_ = 0.73 in 1:1 CH_2_Cl_2_:hexane. ^1^H NMR (δ, ppm in CDCl_3_): 7.35 (*ddt*, *J* = 8.4, 5.3, 2.5 Hz, 8H), 7.04–6.97 (*m*, 8H). ^13^C NMR (δ, ppm in CDCl_3_): 180.4 (*s*), 163.3 (*d*, *J*_FC_ = 251 Hz), 137.3 (*d*, *J* = 3.5 Hz), 130.9 (*d*, *J* = 8.4 Hz), 115.9 (*d*, *J* = 21.8 Hz). ^19^F NMR (δ, ppm in CDCl_3_): (+50.66 relative to C_6_F_6_ inter­nal standard). UV-vis [CH_2_Cl_2_, λ_max_ nm (ɛ_M_, M^−1^·cm^−1^)]: 270 (15,000), 315 (19,100), 600 (90), 860 (12,400). MS (MALDI^+^) Calculated for [C_28_H_16_F_4_S_4_Ni]^+^: *m*/*z* 613.94196; Observed: *m*/*z* 613.842; Error (δ): 163 ppm. Cyclic voltammetry (CH_2_Cl_2_, [^*n*^Bu_4_N][PF_6_] supporting electrolyte, Cp_2_Fe^+^/Cp_2_Fe as reference): **I** + e^−^ → [**I**]^−^, −0.40 V; [**I**]^−^ + e^−^ → [**I**]^2–^, −1.22 V.

## 6. Refinement

Hydrogen atoms were added in calculated positions and refined with isotropic displacement parameters that were 1.2 times those of the carbon atoms to which they were attached. The C—H distance assumed was 0.95 Å. Crystal data, data collection and structure refinement details are summarized in Table 2 ▸.

## Supplementary Material

Crystal structure: contains datablock(s) I, global. DOI: 10.1107/S2056989025007303/jy2064sup1.cif

Structure factors: contains datablock(s) I. DOI: 10.1107/S2056989025007303/jy2064Isup2.hkl

CCDC reference: 2480642

Additional supporting information:  crystallographic information; 3D view; checkCIF report

## Figures and Tables

**Table 1:** Hydrogen-bond geometry (Å, °)

*D*—H⋯*A*	*D*—H	H⋯*A*	*D*⋯*A*	*D*—H⋯*A*
C19—H19⋯F1^i^	1.00 (4)	2.47 (4)	3.136 (5)	123 (3)

Symmetry code: (i) 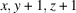.

**Table 2:** Experimental details

Crystal data	
Chemical formula	[Ni(C_14_H_8_F_2_S_2_)_2_]
*M*_r_	615.36
Crystal system, space group	Triclinic, *P* 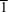
Temperature (K)	150
*a*, *b*, *c* (Å)	9.995 (2), 10.386 (2), 13.958 (3)
α, β, γ (°)	109.61 (3), 90.51 (3), 107.27 (3)
*V* (Å^3^)	1293.7 (5)
*Z*	2
Radiation type	Mo *K*α, λ = 0.71073 Å
μ (mm^−1^)	1.12
Crystal size (mm)	0.09 × 0.07 × 0.03

Data collection	
Diffractometer	Bruker D8
Absorption correction	Multi-scan (*SADABS*; Krause *et al.*, 2015 ▸)
*T*_min_, *T*_max_	0.823, 0.970
No. of measured, independent and observed [*I* > 2σ(*I*)] reflections	31779, 4774, 3330
*R*_int_	0.079

Refinement	
*R*[*F*^2^ > 2σ(*F*^2^)], *wR*(*F*^2^), *S*	0.044, 0.110, 1.05
No. of reflections	4774
No. of parameters	398
H-atom treatment	All H-atom parameters refined
Δρ_max_, Δρ_min_ (e Å^−3^)	0.60, −0.55

Computer programs: *APEX5* and *SAINT* (Bruker, 2024 ▸), *SHELXT2018/2* (Sheldrick, 2015*a* ▸), *SHELXL2019/2* (Sheldrick, 2015*b* ▸) and *SHELXTL* (Sheldrick, 2008 ▸).

## supplementary crystallographic information

### Bis[1,2-bis(4-fluorophenyl)ethylene-1,2-dithiolato(1-)]nickel(II) Crystal data

**Table:**

[Ni(C_14_H_8_F_2_S_2_)_2_]	*Z* = 2
*M**_r_* = 615.36	*F*(000) = 624
Triclinic, *P*1	*D*_x_ = 1.580 Mg m^−^^3^
*a* = 9.995 (2) Å	Mo *K*α radiation, λ = 0.71073 Å
*b* = 10.386 (2) Å	Cell parameters from 6123 reflections
*c* = 13.958 (3) Å	θ = 2.2–24.3°
α = 109.61 (3)°	µ = 1.12 mm^−^^1^
β = 90.51 (3)°	*T* = 150 K
γ = 107.27 (3)°	Prism, black
*V* = 1293.7 (5) Å^3^	0.09 × 0.07 × 0.03 mm

### Bis[1,2-bis(4-fluorophenyl)ethylene-1,2-dithiolato(1-)]nickel(II) Data collection

**Table:**

Bruker D8 diffractometer	4774 independent reflections
Radiation source: sealed tube	3330 reflections with *I* > 2σ(*I*)
Flat graphite monochromator	*R*_int_ = 0.079
Detector resolution: 7.391 pixels mm^-1^	θ_max_ = 25.5°, θ_min_ = 2.5°
ω and φ scans	*h* = −12→12
Absorption correction: multi-scan (*SADABS*; Krause *et al.*, 2015)	*k* = −12→12
*T*_min_ = 0.823, *T*_max_ = 0.970	*l* = −16→16
31779 measured reflections	

### Bis[1,2-bis(4-fluorophenyl)ethylene-1,2-dithiolato(1-)]nickel(II) Refinement

**Table:**

Refinement on *F*^2^	Primary atom site location: Intrinsic Phasing
Least-squares matrix: full	Secondary atom site location: difference Fourier map
*R*[*F*^2^ > 2σ(*F*^2^)] = 0.044	Hydrogen site location: difference Fourier map
*wR*(*F*^2^) = 0.110	All H-atom parameters refined
*S* = 1.05	*w* = 1/[σ^2^(*F*_o_^2^) + (0.0455*P*)^2^ + 0.6394*P*] where *P* = (*F*_o_^2^ + 2*F*_c_^2^)/3
4774 reflections	(Δ/σ)_max_ = 0.001
398 parameters	Δρ_max_ = 0.60 e Å^−^^3^
0 restraints	Δρ_min_ = −0.55 e Å^−^^3^

### Bis[1,2-bis(4-fluorophenyl)ethylene-1,2-dithiolato(1-)]nickel(II) Special details

**Table:**

Geometry. All esds (except the esd in the dihedral angle between two l.s. planes) are estimated using the full covariance matrix. The cell esds are taken into account individually in the estimation of esds in distances, angles and torsion angles; correlations between esds in cell parameters are only used when they are defined by crystal symmetry. An approximate (isotropic) treatment of cell esds is used for estimating esds involving l.s. planes.

### Bis[1,2-bis(4-fluorophenyl)ethylene-1,2-dithiolato(1-)]nickel(II) Fractional atomic coordinates and isotropic or equivalent isotropic displacement parameters (Å^2^)

**Table:**

	*x*	*y*	*z*	*U*_iso_*/*U*_eq_	
Ni1	0.60045 (5)	1.37615 (5)	0.54003 (3)	0.02848 (15)	
S1	0.48356 (9)	1.18073 (10)	0.42242 (7)	0.0296 (2)	
S2	0.79331 (9)	1.32928 (10)	0.51093 (7)	0.0309 (2)	
S3	0.40505 (9)	1.39858 (10)	0.58778 (7)	0.0296 (2)	
S4	0.71238 (9)	1.56654 (10)	0.66330 (7)	0.0292 (2)	
F1	0.3963 (2)	0.5400 (2)	0.08030 (15)	0.0381 (5)	
F2	1.1261 (3)	0.8567 (3)	0.2995 (3)	0.0803 (10)	
F3	−0.0292 (2)	1.5603 (3)	0.90721 (18)	0.0509 (6)	
F4	0.8195 (3)	2.1168 (3)	1.08009 (18)	0.0620 (7)	
C1	0.6030 (4)	1.0919 (4)	0.3789 (3)	0.0295 (8)	
C2	0.7435 (4)	1.1591 (4)	0.4219 (3)	0.0277 (8)	
C3	0.4465 (4)	1.5376 (4)	0.7026 (3)	0.0272 (8)	
C4	0.5882 (4)	1.6177 (4)	0.7369 (3)	0.0286 (8)	
C5	0.5479 (4)	0.9474 (4)	0.2984 (3)	0.0276 (8)	
C6	0.4212 (4)	0.8507 (4)	0.3048 (3)	0.0300 (9)	
H6	0.373 (3)	0.883 (3)	0.365 (3)	0.026 (9)*	
C7	0.3680 (4)	0.7146 (4)	0.2306 (3)	0.0322 (9)	
H7	0.282 (4)	0.653 (4)	0.235 (3)	0.031 (10)*	
C8	0.4452 (4)	0.6772 (4)	0.1513 (3)	0.0305 (9)	
C9	0.5695 (4)	0.7698 (4)	0.1405 (3)	0.0348 (9)	
H9	0.622 (4)	0.748 (4)	0.089 (3)	0.041 (11)*	
C10	0.6201 (4)	0.9057 (4)	0.2136 (3)	0.0343 (9)	
H10	0.705 (4)	0.971 (4)	0.207 (2)	0.025 (9)*	
C11	0.8517 (4)	1.0849 (4)	0.3954 (3)	0.0308 (9)	
C12	0.8265 (4)	0.9498 (4)	0.4025 (3)	0.0348 (9)	
H12	0.749 (4)	0.908 (4)	0.427 (2)	0.021 (9)*	
C13	0.9186 (4)	0.8727 (5)	0.3712 (4)	0.0453 (11)	
H13	0.902 (4)	0.788 (4)	0.375 (3)	0.032 (11)*	
C14	1.0366 (4)	0.9341 (5)	0.3328 (4)	0.0509 (12)	
C15	1.0676 (4)	1.0671 (5)	0.3276 (4)	0.0555 (13)	
H15	1.146 (4)	1.104 (4)	0.304 (3)	0.042 (11)*	
C16	0.9753 (4)	1.1448 (5)	0.3597 (3)	0.0419 (10)	
H16	0.988 (4)	1.229 (4)	0.348 (3)	0.038 (11)*	
C17	0.3250 (3)	1.5554 (4)	0.7605 (3)	0.0276 (8)	
C18	0.3276 (4)	1.5697 (4)	0.8632 (3)	0.0343 (9)	
H18	0.408 (4)	1.568 (3)	0.895 (2)	0.023 (9)*	
C19	0.2092 (4)	1.5709 (5)	0.9130 (3)	0.0375 (10)	
H19	0.206 (4)	1.572 (4)	0.985 (3)	0.035 (10)*	
C20	0.0879 (4)	1.5595 (4)	0.8582 (3)	0.0354 (9)	
C21	0.0799 (4)	1.5467 (4)	0.7578 (3)	0.0337 (9)	
H21	−0.001 (4)	1.543 (4)	0.721 (3)	0.032 (10)*	
C22	0.1993 (4)	1.5444 (4)	0.7088 (3)	0.0319 (9)	
H22	0.189 (4)	1.530 (4)	0.635 (3)	0.039 (10)*	
C23	0.6438 (4)	1.7467 (4)	0.8297 (3)	0.0289 (8)	
C24	0.5873 (4)	1.8608 (4)	0.8526 (3)	0.0339 (9)	
H24	0.511 (5)	1.849 (5)	0.807 (3)	0.061 (14)*	
C25	0.6477 (4)	1.9861 (5)	0.9361 (3)	0.0422 (10)	
H25	0.612 (4)	2.061 (4)	0.945 (3)	0.042 (11)*	
C26	0.7612 (4)	1.9942 (4)	0.9964 (3)	0.0426 (10)	
C27	0.8180 (4)	1.8858 (5)	0.9774 (3)	0.0392 (10)	
H27	0.891 (4)	1.896 (4)	1.017 (3)	0.037 (11)*	
C28	0.7599 (4)	1.7619 (4)	0.8931 (3)	0.0323 (9)	
H28	0.797 (4)	1.683 (4)	0.878 (3)	0.032 (10)*	

### Bis[1,2-bis(4-fluorophenyl)ethylene-1,2-dithiolato(1-)]nickel(II) Atomic displacement parameters (Å^2^)

**Table:**

	*U*^11^	*U*^22^	*U*^33^	*U*^12^	*U*^13^	*U*^23^
Ni1	0.0255 (3)	0.0314 (3)	0.0272 (3)	0.0115 (2)	0.00572 (19)	0.0066 (2)
S1	0.0248 (5)	0.0345 (5)	0.0274 (5)	0.0125 (4)	0.0033 (4)	0.0058 (4)
S2	0.0244 (5)	0.0326 (6)	0.0318 (5)	0.0091 (4)	0.0050 (4)	0.0065 (4)
S3	0.0240 (5)	0.0328 (5)	0.0282 (5)	0.0098 (4)	0.0041 (4)	0.0054 (4)
S4	0.0239 (5)	0.0308 (5)	0.0308 (5)	0.0102 (4)	0.0063 (4)	0.0071 (4)
F1	0.0403 (12)	0.0353 (13)	0.0334 (12)	0.0142 (10)	−0.0010 (10)	0.0035 (10)
F2	0.0407 (15)	0.0612 (18)	0.144 (3)	0.0355 (14)	0.0282 (16)	0.0254 (18)
F3	0.0303 (12)	0.0822 (19)	0.0542 (15)	0.0280 (12)	0.0184 (11)	0.0323 (14)
F4	0.0683 (17)	0.0385 (15)	0.0509 (16)	0.0048 (13)	−0.0035 (13)	−0.0083 (12)
C1	0.032 (2)	0.037 (2)	0.0262 (19)	0.0194 (18)	0.0090 (16)	0.0125 (17)
C2	0.031 (2)	0.028 (2)	0.029 (2)	0.0132 (17)	0.0121 (16)	0.0135 (17)
C3	0.031 (2)	0.028 (2)	0.030 (2)	0.0171 (16)	0.0059 (16)	0.0114 (17)
C4	0.033 (2)	0.030 (2)	0.028 (2)	0.0157 (17)	0.0091 (16)	0.0120 (17)
C5	0.0238 (18)	0.035 (2)	0.027 (2)	0.0138 (16)	0.0039 (15)	0.0104 (17)
C6	0.029 (2)	0.038 (2)	0.026 (2)	0.0152 (18)	0.0072 (17)	0.0094 (18)
C7	0.027 (2)	0.033 (2)	0.039 (2)	0.0111 (18)	0.0042 (18)	0.0138 (19)
C8	0.034 (2)	0.031 (2)	0.026 (2)	0.0154 (17)	−0.0035 (17)	0.0045 (17)
C9	0.033 (2)	0.037 (2)	0.031 (2)	0.0149 (19)	0.0077 (18)	0.0051 (19)
C10	0.029 (2)	0.038 (2)	0.034 (2)	0.0071 (19)	0.0067 (18)	0.0126 (19)
C11	0.0225 (19)	0.035 (2)	0.031 (2)	0.0097 (16)	0.0007 (16)	0.0067 (17)
C12	0.025 (2)	0.035 (2)	0.045 (2)	0.0079 (18)	0.0046 (18)	0.016 (2)
C13	0.034 (2)	0.033 (3)	0.070 (3)	0.013 (2)	0.000 (2)	0.018 (2)
C14	0.027 (2)	0.045 (3)	0.078 (3)	0.021 (2)	0.006 (2)	0.011 (2)
C15	0.027 (2)	0.053 (3)	0.090 (4)	0.015 (2)	0.025 (2)	0.028 (3)
C16	0.031 (2)	0.033 (2)	0.064 (3)	0.0116 (19)	0.013 (2)	0.019 (2)
C17	0.0242 (19)	0.025 (2)	0.034 (2)	0.0089 (16)	0.0034 (16)	0.0100 (17)
C18	0.027 (2)	0.050 (3)	0.033 (2)	0.0179 (19)	0.0059 (17)	0.0184 (19)
C19	0.034 (2)	0.055 (3)	0.034 (2)	0.022 (2)	0.0109 (18)	0.022 (2)
C20	0.024 (2)	0.045 (3)	0.044 (2)	0.0175 (18)	0.0124 (18)	0.018 (2)
C21	0.022 (2)	0.046 (3)	0.036 (2)	0.0152 (18)	0.0001 (17)	0.0137 (19)
C22	0.032 (2)	0.039 (2)	0.025 (2)	0.0125 (18)	0.0016 (17)	0.0108 (18)
C23	0.0255 (19)	0.034 (2)	0.030 (2)	0.0108 (16)	0.0083 (16)	0.0131 (17)
C24	0.032 (2)	0.036 (2)	0.035 (2)	0.0146 (19)	0.0055 (18)	0.0105 (19)
C25	0.045 (3)	0.034 (3)	0.047 (3)	0.018 (2)	0.013 (2)	0.009 (2)
C26	0.043 (2)	0.036 (2)	0.034 (2)	0.004 (2)	0.004 (2)	0.0021 (19)
C27	0.027 (2)	0.043 (3)	0.040 (3)	0.0033 (19)	−0.0017 (19)	0.012 (2)
C28	0.025 (2)	0.032 (2)	0.038 (2)	0.0091 (18)	0.0068 (17)	0.0107 (19)

### Bis[1,2-bis(4-fluorophenyl)ethylene-1,2-dithiolato(1-)]nickel(II) Geometric parameters (Å, º)

**Table:**

Ni1—S4	2.1189 (15)	C11—C12	1.388 (5)
Ni1—S1	2.1213 (15)	C12—C13	1.374 (5)
Ni1—S3	2.1217 (11)	C12—H12	0.90 (3)
Ni1—S2	2.1344 (11)	C13—C14	1.372 (6)
S1—C1	1.714 (3)	C13—H13	0.86 (4)
S2—C2	1.705 (4)	C14—C15	1.349 (6)
S3—C3	1.704 (4)	C15—C16	1.382 (6)
S4—C4	1.710 (3)	C15—H15	0.89 (4)
F1—C8	1.372 (4)	C16—H16	0.91 (4)
F2—C14	1.358 (4)	C17—C18	1.390 (5)
F3—C20	1.361 (4)	C17—C22	1.398 (5)
F4—C26	1.369 (4)	C18—C19	1.379 (5)
C1—C2	1.393 (5)	C18—H18	0.92 (3)
C1—C5	1.478 (5)	C19—C20	1.380 (5)
C2—C11	1.487 (5)	C19—H19	1.00 (4)
C3—C4	1.396 (5)	C20—C21	1.362 (5)
C3—C17	1.488 (5)	C21—C22	1.382 (5)
C4—C23	1.470 (5)	C21—H21	0.94 (4)
C5—C6	1.388 (5)	C22—H22	0.99 (4)
C5—C10	1.402 (5)	C23—C28	1.391 (5)
C6—C7	1.383 (5)	C23—C24	1.403 (5)
C6—H6	0.98 (3)	C24—C25	1.386 (6)
C7—C8	1.370 (5)	C24—H24	0.94 (4)
C7—H7	0.92 (4)	C25—C26	1.371 (6)
C8—C9	1.372 (5)	C25—H25	0.92 (4)
C9—C10	1.374 (5)	C26—C27	1.357 (6)
C9—H9	0.91 (4)	C27—C28	1.380 (5)
C10—H10	0.94 (3)	C27—H27	0.87 (4)
C11—C16	1.387 (5)	C28—H28	0.96 (4)
			
S4—Ni1—S1	176.95 (4)	C14—C13—H13	122 (2)
S4—Ni1—S3	90.79 (5)	C12—C13—H13	121 (2)
S1—Ni1—S3	87.71 (5)	C15—C14—F2	119.2 (4)
S4—Ni1—S2	89.36 (5)	C15—C14—C13	123.0 (4)
S1—Ni1—S2	91.59 (5)	F2—C14—C13	117.8 (4)
S3—Ni1—S2	168.77 (4)	C14—C15—C16	119.2 (4)
C1—S1—Ni1	105.36 (13)	C14—C15—H15	120 (3)
C2—S2—Ni1	104.64 (13)	C16—C15—H15	120 (3)
C3—S3—Ni1	105.74 (13)	C15—C16—C11	119.9 (4)
C4—S4—Ni1	106.05 (14)	C15—C16—H16	120 (2)
C2—C1—C5	124.8 (3)	C11—C16—H16	120 (2)
C2—C1—S1	118.4 (3)	C18—C17—C22	118.3 (3)
C5—C1—S1	116.9 (3)	C18—C17—C3	121.9 (3)
C1—C2—C11	121.9 (3)	C22—C17—C3	119.6 (3)
C1—C2—S2	119.6 (3)	C19—C18—C17	121.2 (4)
C11—C2—S2	118.4 (3)	C19—C18—H18	120 (2)
C4—C3—C17	125.9 (3)	C17—C18—H18	118 (2)
C4—C3—S3	118.8 (3)	C18—C19—C20	118.0 (4)
C17—C3—S3	115.2 (3)	C18—C19—H19	122 (2)
C3—C4—C23	126.6 (3)	C20—C19—H19	119 (2)
C3—C4—S4	118.0 (3)	C21—C20—F3	118.8 (3)
C23—C4—S4	115.3 (3)	C21—C20—C19	123.1 (3)
C6—C5—C10	118.6 (3)	F3—C20—C19	118.1 (3)
C6—C5—C1	120.1 (3)	C20—C21—C22	118.1 (3)
C10—C5—C1	121.2 (3)	C20—C21—H21	124 (2)
C7—C6—C5	121.1 (3)	C22—C21—H21	118 (2)
C7—C6—H6	122 (2)	C21—C22—C17	121.2 (3)
C5—C6—H6	116.9 (19)	C21—C22—H22	116 (2)
C8—C7—C6	118.0 (4)	C17—C22—H22	122 (2)
C8—C7—H7	122 (2)	C28—C23—C24	118.6 (4)
C6—C7—H7	120 (2)	C28—C23—C4	120.3 (3)
F1—C8—C7	118.1 (3)	C24—C23—C4	121.0 (3)
F1—C8—C9	118.8 (3)	C25—C24—C23	120.3 (4)
C7—C8—C9	123.1 (4)	C25—C24—H24	123 (3)
C8—C9—C10	118.5 (4)	C23—C24—H24	117 (3)
C8—C9—H9	125 (2)	C26—C25—C24	118.4 (4)
C10—C9—H9	117 (2)	C26—C25—H25	124 (3)
C9—C10—C5	120.7 (4)	C24—C25—H25	117 (3)
C9—C10—H10	119 (2)	C27—C26—F4	118.3 (4)
C5—C10—H10	120 (2)	C27—C26—C25	123.0 (4)
C16—C11—C12	118.9 (4)	F4—C26—C25	118.6 (4)
C16—C11—C2	121.6 (4)	C26—C27—C28	118.7 (4)
C12—C11—C2	119.5 (3)	C26—C27—H27	120 (3)
C13—C12—C11	121.3 (4)	C28—C27—H27	122 (3)
C13—C12—H12	117 (2)	C27—C28—C23	120.9 (4)
C11—C12—H12	122 (2)	C27—C28—H28	121 (2)
C14—C13—C12	117.6 (4)	C23—C28—H28	118 (2)
			
Ni1—S1—C1—C2	−1.9 (3)	C11—C12—C13—C14	0.0 (6)
Ni1—S1—C1—C5	177.5 (2)	C12—C13—C14—C15	2.2 (7)
C5—C1—C2—C11	−4.8 (6)	C12—C13—C14—F2	−178.4 (4)
S1—C1—C2—C11	174.6 (3)	F2—C14—C15—C16	178.9 (4)
C5—C1—C2—S2	177.5 (3)	C13—C14—C15—C16	−1.8 (8)
S1—C1—C2—S2	−3.1 (4)	C14—C15—C16—C11	−0.9 (7)
Ni1—S2—C2—C1	6.4 (3)	C12—C11—C16—C15	3.0 (6)
Ni1—S2—C2—C11	−171.4 (3)	C2—C11—C16—C15	−174.5 (4)
Ni1—S3—C3—C4	7.3 (3)	C4—C3—C17—C18	−45.4 (5)
Ni1—S3—C3—C17	−168.9 (2)	S3—C3—C17—C18	130.4 (3)
C17—C3—C4—C23	−8.8 (6)	C4—C3—C17—C22	140.6 (4)
S3—C3—C4—C23	175.5 (3)	S3—C3—C17—C22	−43.6 (4)
C17—C3—C4—S4	173.2 (3)	C22—C17—C18—C19	1.0 (6)
S3—C3—C4—S4	−2.5 (4)	C3—C17—C18—C19	−173.1 (4)
Ni1—S4—C4—C3	−3.7 (3)	C17—C18—C19—C20	−0.9 (6)
Ni1—S4—C4—C23	178.1 (2)	C18—C19—C20—C21	0.3 (6)
C2—C1—C5—C6	136.9 (4)	C18—C19—C20—F3	179.8 (3)
S1—C1—C5—C6	−42.4 (4)	F3—C20—C21—C22	−179.3 (3)
C2—C1—C5—C10	−44.1 (5)	C19—C20—C21—C22	0.3 (6)
S1—C1—C5—C10	136.6 (3)	C20—C21—C22—C17	−0.2 (6)
C10—C5—C6—C7	1.3 (5)	C18—C17—C22—C21	−0.4 (6)
C1—C5—C6—C7	−179.6 (3)	C3—C17—C22—C21	173.8 (3)
C5—C6—C7—C8	1.0 (6)	C3—C4—C23—C28	136.3 (4)
C6—C7—C8—F1	176.8 (3)	S4—C4—C23—C28	−45.6 (4)
C6—C7—C8—C9	−2.2 (6)	C3—C4—C23—C24	−48.0 (5)
F1—C8—C9—C10	−178.0 (3)	S4—C4—C23—C24	130.1 (3)
C7—C8—C9—C10	1.0 (6)	C28—C23—C24—C25	0.7 (6)
C8—C9—C10—C5	1.4 (6)	C4—C23—C24—C25	−175.1 (4)
C6—C5—C10—C9	−2.6 (6)	C23—C24—C25—C26	−1.3 (6)
C1—C5—C10—C9	178.4 (3)	C24—C25—C26—C27	0.6 (7)
C1—C2—C11—C16	125.5 (4)	C24—C25—C26—F4	−178.8 (4)
S2—C2—C11—C16	−56.8 (5)	F4—C26—C27—C28	−180.0 (3)
C1—C2—C11—C12	−51.9 (5)	C25—C26—C27—C28	0.6 (7)
S2—C2—C11—C12	125.8 (3)	C26—C27—C28—C23	−1.2 (6)
C16—C11—C12—C13	−2.6 (6)	C24—C23—C28—C27	0.6 (6)
C2—C11—C12—C13	174.9 (4)	C4—C23—C28—C27	176.4 (3)

### Bis[1,2-bis(4-fluorophenyl)ethylene-1,2-dithiolato(1-)]nickel(II) Hydrogen-bond geometry (Å, º)

**Table:**

*D*—H···*A*	*D*—H	H···*A*	*D*···*A*	*D*—H···*A*
C19—H19···F1^i^	1.00 (4)	2.47 (4)	3.136 (5)	123 (3)

Symmetry code: (i) *x*, *y*+1, *z*+1.
